# The role of *Curcuma longa* essential oil in controlling acute toxoplasmosis by improving the immune system and reducing inflammation and oxidative stress

**DOI:** 10.3389/fcimb.2023.1161133

**Published:** 2023-04-27

**Authors:** Fatemeh Ezzatkhah, Hossein Mahmoudvand, Yosra Raziani

**Affiliations:** ^1^ Department of Laboratory Sciences, Sirjan School of Medical Sciences, Sirjan, Iran; ^2^ Razi Herbal Medicines Research Center, Lorestan University of Medical Sciences, Khorramabad, Iran; ^3^ Nursing Department, Al-Mustaqbal University College, Hillah, Babylon, Iraq

**Keywords:** toxoplasmosis, RH strain, prophylaxis, essential oil, tachyzoites

## Abstract

**Background:**

Chemotherapy with synthetic drugs is the principal approach for toxoplasmosis treatment; however, recent studies reported the limitations and adverse side effects of these chemical drugs.

**Objective:**

This study aimed to examine the *in vitro* and *in vivo* effects of *Curcuma longa* essential oil (CLE) against the *Toxoplasma gondii* RH strain.

**Methods:**

The *in vitro* effect of different concentrations of CLE on *T. gondii* tachyzoites was assessed by cell viability assay. Flow cytometry and apoptosis analysis were performed, and nitric oxide production by CLE was also evaluated in tachyzoites. BALB/c mice were orally treated with various doses (1.25, 2.5, and 5 mg·kg^−1^·day^−1^) of CLE for 2 weeks. After the induction of acute toxoplasmosis in the mice, their survival rate and the mean number of peritoneal parasites were checked. The hepatic level of antioxidant enzymes and oxidative stress markers was evaluated by commercial kits. The mRNA expression level of proinflammatory cytokines such as interleukin 1-beta (IL-1β) and interferon-gamma (IFN-γ) was evaluated by quantitative real-time PCR.

**Results:**

CLE, especially at 50 µg/ml, showed potent inhibitory effects on *T. gondii* tachyzoites. It increased the survival rate (ninth day) and reduced the mean number of peritoneal tachyzoites in the infected mice. CLE dependently increased (p < 0.01) the number of necrotic and apoptotic cells as well as NO production. CLE significantly (p < 0.05) reduced the hepatic level of oxidative stress markers but increased (p < 0.001) the antioxidant enzymes and proinflammatory cytokines in the infected mice, with no important toxicity for vital organs.

**Conclusion:**

The findings of this survey revealed the significant *in vitro* inhibitory effects of CLE on *T. gondii* tachyzoites. The results also exhibited promising *in vivo* effects of CLE. CLE improved the survival rate of infected mice and reduced the parasite number in them. Although the mechanisms of action of CLE are not clear, our study demonstrated its beneficial effects on acute toxoplasmosis by strengthening the immune system and reducing inflammation and oxidative stress. Still, more studies are required to confirm these results.

## Introduction

A highly frequent infection worldwide that infects a wide range of warm-blooded animals and humans is toxoplasmosis, caused by the *Toxoplasma gondii* parasite (Saadatnia and Golkar, 2012). Based on seroepidemiological studies, nearly 30% of the global population has anti-*T. gondii* antibodies, suggesting that they were infected with *T. gondii* (Molan et al., 2019). The main ways whereby humans contract toxoplasmosis include the consumption of food and water contaminated with oocysts defecated by cats (definitive host) or through ingestion of raw meat having tissue cysts (Martinez et al., 2018). However, congenital toxoplasmosis can be observed *via* the placental spread of parasites during pregnancy (Martinez et al., 2018). Concerning the clinical symptoms of toxoplasmosis, acquired toxoplasmosis in healthy people is often asymptomatic and rarely causes severe manifestations; nevertheless, in people with a defective immune system, e.g., patients with AIDS and organ recipients, it is accompanied by severe symptoms and complications (Dubey et al., 2012).

Today, due to the absence of an effective vaccine for preventing toxoplasmosis, prophylactic programs in some people (e.g., immunocompromised patients and pregnant women who were seronegative for toxoplasmosis) are strongly recommended (Wang et al., 2017). Chemotherapy with synthetic drugs (e.g., pyrimethamine, sulfadiazine, spiramycin, and atovaquone) is the principal approach for the treatment of acquired and congenital toxoplasmosis (Martina et al., 2011). Several studies reported the limitations and adverse side effects of these chemical drugs, e.g., hematuria, hypersensitivity reactions, thrombocytopenia, leukopenia, megaloblastic anemia, teratogenic effects, and renal complications (Gajurel et al., 2015). This motivated the researchers to find an effective agent with negligible side effects and ideal effectiveness for toxoplasmosis treatment.

Due to their few side effects and favorable acceptance by patients, medicinal plants and their derived compounds are broadly used for the treatment and prevention of various diseases (Cheraghipour et al., 2021). Reviews have shown that crude herb materials, including extracts, essential oils, and isolated components, are considered to prevent the spread and exacerbation of numerous foodborne infections (McFarland et al., 2016). Recently, the anti-*Toxoplasma* effects of various medicinal herbs, e.g., *Zataria multiflora*, *Eurycoma longifolia*, *Zingiber officinale*, *Myrtus communis*, *Berberis vulgaris*, *Sophora flavescens*, *Nigella sativa*, *Piper betle*, and *Allium cepa*, have been investigated (Montazeri et al., 2017). Nevertheless, final approval for the use of these herbs for toxoplasmosis therapy has been suspended due to some limitations.

Turmeric or *Curcuma longa* L. (Zingiberaceae family) is a well-known spice usually used in foods and traditional medicine (Verma et al., 2018). The herb in traditional medicine has been applied for treating and controlling some diseases, e.g., gastrointestinal disorders, microbial infections (e.g., cold, bronchitis, fever, and infection of the eye, urinary tract, and lung), cardiac illnesses, nausea, and diarrhea (Jyotirmayee and Mahalik, 2022).

Recent studies showed that *C. longa* displayed numerous pharmacological properties, e.g., antidiabetic, antinociceptive, anti-inflammatory, anticancer, and antimicrobial features (Verma et al., 2018; Chanda and Ramachandra, 2019; Jyotirmayee and Mahalik, 2022). Turmeric has an essential oil composed of valeric, caprylic, and phenolic acids and also contains sabinene, cineol, borneol, tourmerol alcohol, and curcumin (which is responsible for the yellow color of turmeric) (Niranjan and Prakash, 2008). In addition to being genetically controlled, the amount and composition of the essential oil also depend on the geographical location of the plant, climatic situations at the time of seed formation and ripening, harvest time, harvest season, and the essential oil extraction method (Saedi Dezaki et al., 2016). Based on the potential therapeutic benefits of *C. longa*, the present study aimed to evaluate the *in vitro* and *in vivo* effects of *C. longa* essential oil (CLE) against the *T. gondii* RH strain.

## Materials and methods

### Plant collections

The rhizome of turmeric was purchased in June 2022 from a store in Kerman province, Iran. After identification by a botanist, a voucher example was kept at the Herbarium section of Pharmaceutics Research Center, Kerman, Iran (no. 56658).

### Isolation and chemical composition of essential oil

For the experiment, 250 g of dried and powdered rhizome was used for hydro-distillation technique by the Clevenger-type apparatus. After dehydration of the attained essential oil with anhydrous sodium sulfate, it was stored at 4°C until use (Kareshk et al., 2015). To study the chemical composition of essential oil, a Hewlett-Packard 6890 (Palo Alto, CA, USA) equipped with an HP-5MS column (30 m × 0.25 mm, film thickness 0.25 mm) was used based on the conditions reported previously (Mahmoudvand et al., 2020). The compounds were recognized based on the assessment of their mass spectra with those of the National Institute of Standards and Technology (NIST) mass spectral library (NIST 2014) and those reported by Adams or with literature values (NIST, 2014).

### Parasites

In this study, *T. gondii* RH tachyzoites prepared by the Kerman University of Medical Science, Kerman, Iran, were used. Parasites were intraperitoneally (IP) passaged in BALB/c mice. Then, the collected peritoneal tachyzoites were washed with PBS (pH 7.4) and adjusted into 1 × 10^4^ tachyzoites/ml experiments.

### Cell culture

J774-A1 cells were prepared from Pasture Institute, Iran, and cultured in RPMI-1640 medium supplemented with 10% inactivated fetal bovine serum (FBS) and pen/strep (100 units/ml) and maintained at 37°C with 5% CO_2._

### *In vitro* inhibitory effects on *T. gondii* tachyzoites

To study the *in vitro* inhibitory effects of CLE on *T. gondii* tachyzoites, 300 µl of tachyzoites (1 × 10^6^ cells/ml) was exposed to CLE and atovaquone (12.5–50 µg/ml) for 0.5–3 h at 37°C, whereas the selection of these concentrations was based on the primary experiments. Then, 50 µl of MTT (3-(4,5-dimethylthiazol-2-yl)-2,5-diphenyltetrazolium bromide, 5 mg/ml) was put in the tested tubes and kept warm at 37°C for 4 h with 5% CO_2_. Dimethyl sulfoxide (DMSO) was added to the tubes to stop the reaction. Finally, the optical density (OD) of the samples was recorded at 575 nm by an ELISA reader (ELx800; BioTek, Winooski, VT, USA) (Teimouri et al., 2018). The negative control was tachyzoites treated with normal saline + Tween 20. All the experiments were performed in triplicate.

### Flow cytometry and apoptosis analysis

In order to quantitatively determine the amount of apoptosis of cells, an apoptosis detection kit (Sigma-Aldrich, Taufkirchen, Germany) was used according to the manufacturer’s instructions; the cells and parasites were stained by the two factors FITC-V Annexin and propidium iodide (PI) and were analyzed using the Sysmex flow cytometry device. Briefly, tachyzoites (1 × 10^6^ cells/ml) were exposed to CLE (12.5–50 µg/ml) for 24 h, and the treated parasites were washed with culture medium and phosphate-buffered saline. Then Annexin buffer and FITC-V Annexin conjugate were added. After the addition of propidium iodine solution and incubation for 10 min in a dark place, the level of apoptotic and necrotic cells was determined by a Facscalibur™ Flow Cytometer BD.

### Effect on infectivity and intracellular parasites

First, cells (1 × 10^5^) were planted in a 96-well cell plate and kept warm at 37°C for 24 h. Then, cells in each well were exposed to 5 × 10^5^ tachyzoites for 24 h. After the supernatant was discarded, artifacts were removed by washing with sterile PBS. Next, the cells were infected by *T. gondii* and exposed to CLE and atovaquone (12.5–50 µg/ml) for 48 h. The treated cells were smeared and stained *via* Giemsa and then checked by a light microscope to evaluate the infectivity rate and mean number of parasites in 100 examined cells. The 50% inhibitory concentration (IC_50_) values were also calculated by Probit test in SPSS software ver. 25.0 (Teimouri et al., 2018). All the experiments were performed in triplicate.

### Effect of nitric oxide production

The effect of CLE on nitric oxide (NO) creation in macrophage cells was studied using the NO colorimetric kit (Sigma-Aldrich, Germany) according to the manufacturer’s instructions. Cells (1 × 10^6^ cells/ml) were exposed to CLE (12.5–50 µg/ml) for 48 h. Then, supernatants were collected and transferred into a 96-well microplate. Subsequently, Griess reagents A and B (60 μl) were added to wells, with the absorbance of the plates at 540 nm in an ELISA reader (BioTek-ELx800). Non-treated cells and cells treated with lipopolysaccharide (LPS) + interferon-gamma (IFN-γ) were considered negative and positive controls, respectively.

### *In vivo* effects of CLE on the animal model of toxoplasmosis

#### Animals

A total of 84 BALB/c mice aged 40–60 days (20–25 g) were included in this survey. The mice were housed in optimum conditions and heat, with *ad libitum* supplies of food and water. The animal experiments were performed in line with the procedures for the Care and Use of Laboratory Animals.

### Ethics

The Sirjan Faculty of Medical Sciences approved this study (No. IR.SIRUMS.REC.1400.007).

### Study design

Sixty mice were allocated to five groups (12 mice each), namely orally administrated normal saline, atovaquone 100 mg·kg^−1^·day^−1^, and CLE 1.25, 2.5, and 5 mg·kg^−1^·day^−1^ for 14 days; the selection of these concentrations was based on the primary experiments. Fifteen days after starting the treatment, the mice in all groups were infected intraperitoneally with parasite solution (100 µl of tachyzoites (1 × 10^4^ tachyzoites/ml) mixed with 100 µl of sterile normal saline).

### Survival rate and parasitological tests

The treated animals were daily observed, and the level of survival was noted in the animals in each group. On the third day, after the peritoneal fluids of the tested mice were collected, the number of peritoneal parasites of the mice was microscopically checked (Shakibaie et al., 2020).

### Assessment of oxidative stress indicators

On the third day post-toxoplasmosis induction, six mice from each group were euthanized (by intraperitoneal injection of sodium pentobarbital) to study oxidative stress factors, antioxidant enzymes, and proinflammatory cytokines. The hepatic levels of lipid peroxidation (LPO) and NO in liver homogenates were determined by a biodiagnostic commercial kit (Pars Azmon, Tehran, Iran) based on the kits’ instructions.

### Assessment of antioxidant indicators

The levels of some enzymes involved in antioxidant mechanisms such as glutathione peroxidase (GPx) and superoxide dismutase (SOD) enzyme activity were evaluated using commercial kits (Pars Azmon, Iran) based on the kits’ instructions.

### Evaluating proinflammatory cytokines

The serum level of proinflammatory cytokines, e.g., IFN-γ and interleukin 1-beta (IL-1β), was measured *via* a Mouse IFN-gamma and IL-1 beta ELISA kit (Pars Azmon, Iran) according to the manufacturer’s protocols.

### Proinflammatory cytokines’ mRNA expression

The mRNA expression level of IL-1β and IFN-γ was assessed by real-time PCR. Whole RNA was obtained *via* an RNeasy tissue kit (Qiagen, Hilden, Germany) as explained by the manufacturer’s instructions. The complementary DNA (cDNA) was synthesized by cDNA Synthesis Kits (Qiagen, Germany) according to the manufacturer’s guidelines. Sequence primers applied in real-time PCR are shown in **Table 1**. The thermal program of the procedure was 97°C for 6 min, 40 cycles of 97°C for 12 s, followed by 57°C for 35 s. The 2^−ΔΔCT^ method *via* the iQTM5 optical system software (Bio-Rad, Hercules, CA, USA) was used to measure the expression level. The housekeeping gene and normalization control were β-actin (Albalawi et al., 2021).

**Table 1:** The primers applied for real-time PCR.

Amplicon	Primers	Sequence (5′–3′)	Size (bp)
IL-1β	F R	AACCTGCTGGTGTGTGACGTTC CAGCACGAGGCTTTTTTGTTGT	78
IFN-γ	F R	ATGAACGCTACACACTGCATC CCATCCTTTTGCCAGTTCCTC	182
β-Actin	F R	GTGACGTTGACATCCGTAAAGA GCCGGACTCATCGTACTCC	245

### Toxicity properties of CLE on vital organ function

A total of 24 healthy mice in four groups were treated orally with normal saline and CLE at doses of 1.25-5 mg·kg^−1^·day^−1^ for 2 weeks. One day after the treatment, blood samples from all the tested mice were collected by cardiac puncture. After centrifuging the samples at 4,000 rpm for 10 min, the obtained sera were stored at −20°C until use (Shakibaie et al., 2013). Then, the serum levels of alanine aminotransferase (ALT), aspartate aminotransferase (AST), alkaline phosphatase (ALP), creatinine (Cr), and blood urea nitrogen (BUN) were calculated *via* commercial kits (Pars Azmon, Iran).

### Statistical analysis

The data were represented as means ± standard deviation and were analyzed in SPSS 22.0 (SPSS Inc., Chicago, IL, USA). One-way analysis of variance (ANOVA) with Tukey’s *post-hoc* test was applied to compare the differences among the groups.

## Results

### Chemical composition of CLE

The yield of yellow essential oil was 2.76% (v/w). As shown in **Table 2**, 22 constituents were recognized in CLE, which account for about 95.5% of this oil. The main components were β-turmerone (21.8%), Ar-turmerone (14.7%), and α-turmerone (12.4).

**Table 2:** GC/MS analysis of chemical compositions of *Curcuma longa* essential oil.

No	Components	KI^a^	% Composition
1.	α-Thujene	7.434	0.7
2.	α-Pinene	7.66	0.5
3.	β-Myrcene	9.48	0.8
4.	l-Phellandrene	9.97	8.8
5.	ρ-Cymene	10.47	5.4
6.	1,8-Cineole	10.72	3.7
7.	Limonene	10.76	0.5
8.	α-Terpinolene	12.68	0.8
9.	*trans*-Caryophyllene	22.43	1.8
10.	α-Caryophyllene	23.25	0.5
11.	α-Curcumene	23.84	4.4
12.	α-Zingiberene	24.25	3.2
13.	β-Bisabolene	24.58	0.9
14.	β-Sesquiphellandrene	24.94	4.2
15.	2-Phenyl-1-D1-Aziridine	25.87	1.47
16.	β-Caryophyllene	26.12	0.9
17.	β-Atlantone	26.25	0.9
18.	1,3,5-Cycloheptatriene	26.35	1.4
19.	Pyrazine	26.45	0.7
20.	β-Bisabolene	26.87	0.8
21.	γ-Curcumene	27.23	2.1
22.	β-Turmerone	27.90	21.8
23.	Ar-Turmerone	28.10	14.7
24.	α-Turmerone	28.74	12.4.1
25.	3-Fluorophenyl isocyanate	29.91	0.5
	α-Atlantone	30.20	1.6
	Total		95.5

GC/MS, gas chromatography/mass spectrometry.

^a^Kovats index on non-polar DB-5 MS column in reference to n-alkanes.

### *In vitro* inhibitory effects on *T. gondii* tachyzoites

**Figure 1:**
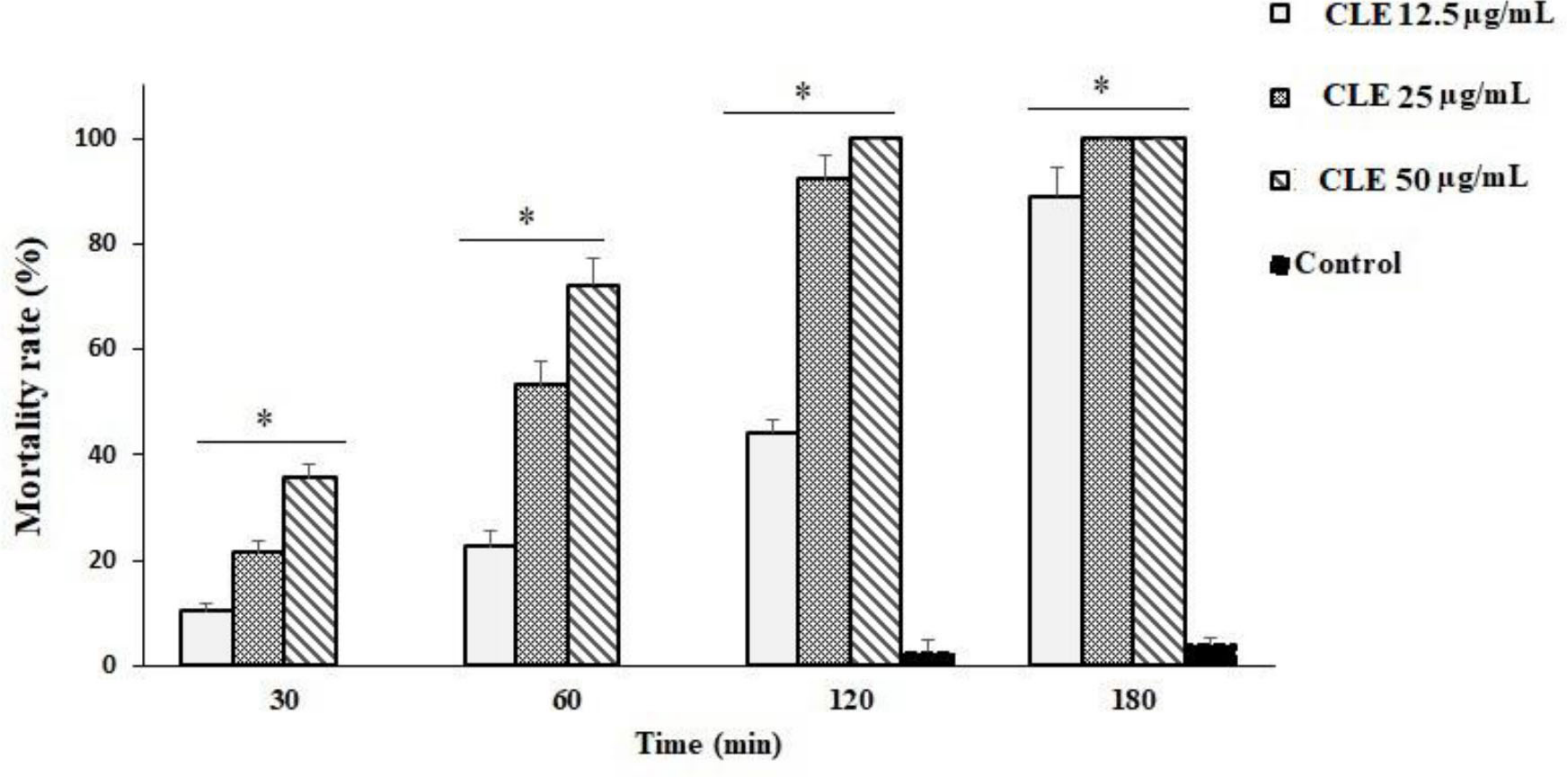
Effects of *Curcuma longa* essential oil (CLE) against *Toxoplasma gondii* tachyzoites after 0.5, 1, 2, and 3 h of incubation in comparison with the control group. Data are expressed as the mean ± SD (n = 3). *p < 0.001 compared with the control group.

As depicted in **Figure 1**, various concentrations of CLE demonstrated significant (p < 0.001) *in vitro* inhibitory effects on the growth rate of parasites after 0.5–3 h of incubation when compared with the control group. CLE at 25 and 50 µg/ml caused 100% mortality in tachyzoites after 3 and 2 h of incubation, respectively.

### Flow cytometry and apoptosis analysis

**Figure 2:**
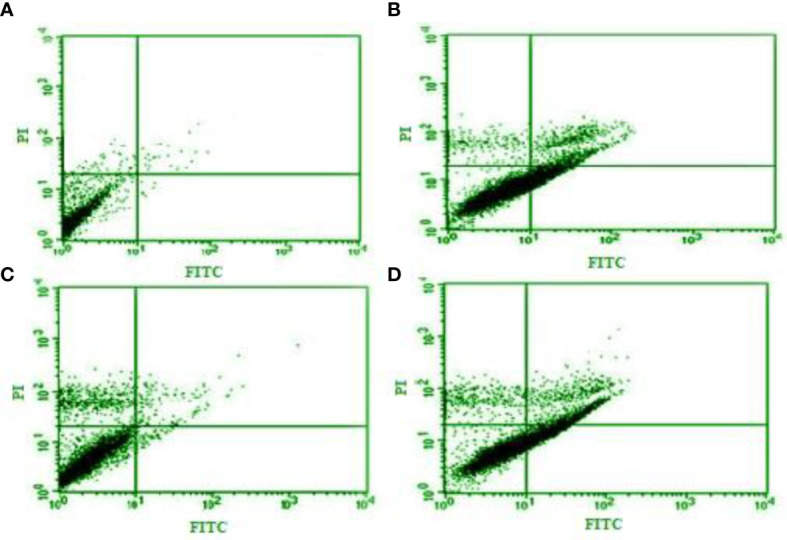
Flow cytometry and apoptosis analysis of *Curcuma longa* essential oil at 12.5, 25, and 50 µg/ml **(B–D)** on the apoptotic and necrotic cells in tachyzoites of *Toxoplasma gondii* compared with control group **(A)**.

The flow cytometry and apoptosis analysis exhibited that this essential oil dose dependently increased (p < 0.01) the number of necrotic (from 0.25% to 10.1%) and apoptotic cells (from 1.6% to 31.3%), respectively (**Figure 2**).

### Evaluating infectivity, intracellular parasites, and cytotoxicity

**Figure 3:**
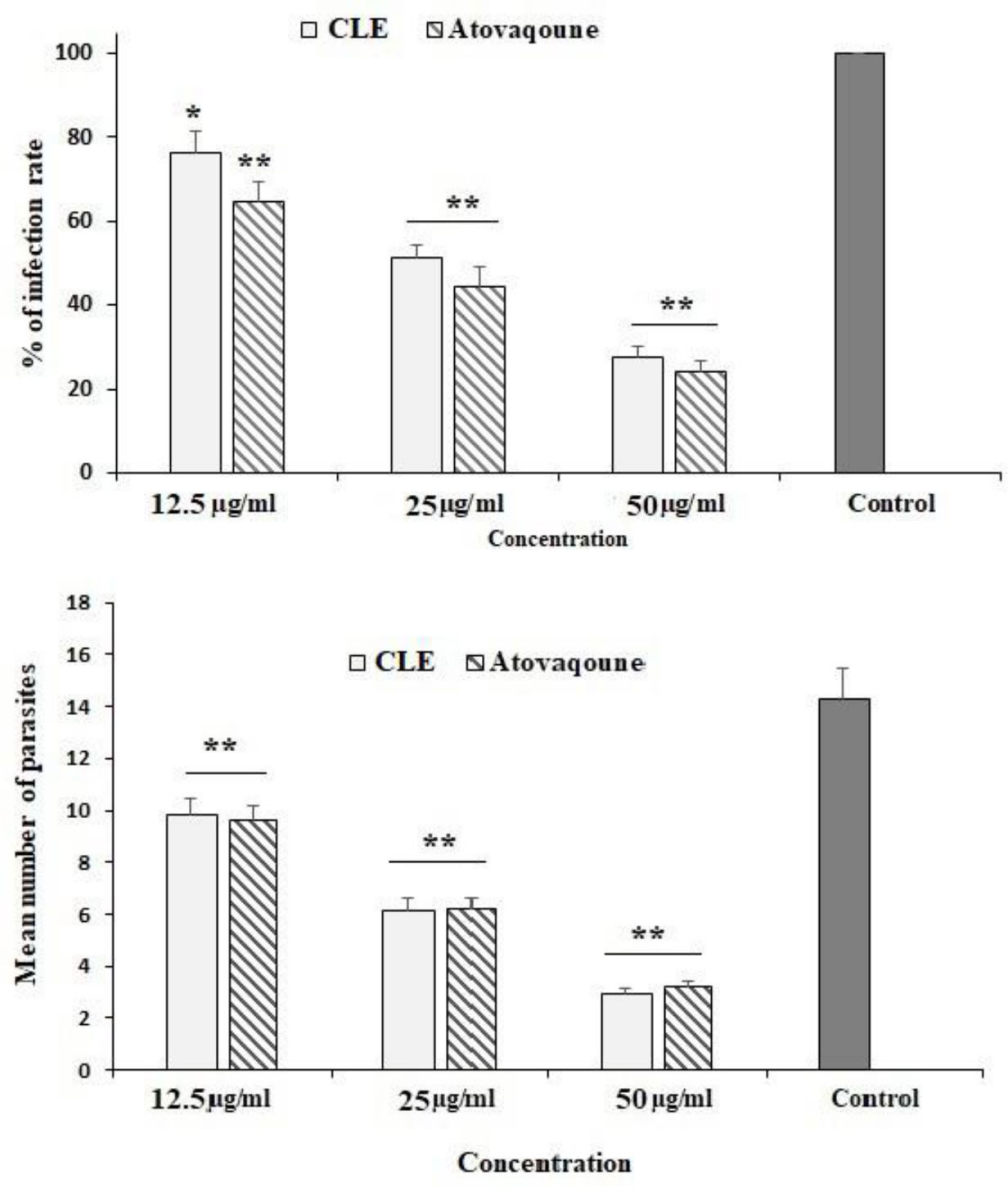
The infection rate of the Vero cells infected with *Toxoplasma gondii* tachyzoites after exposure to *Curcuma longa* essential oil (CLE) at 12.5, 25, and 50 µg/ml for 24 h. The intracellular replication of *T. gondii* in infected Vero cells after treatment with various concentrations of CLE. Data are expressed as the mean ± SD (n = 3). *p < 0.05; **p < 0.05.

The maximum inhibitory activity of CLE on the infectivity of *T. gondii*-infected Vero cells was observed at 50 µg/ml, where it significantly declined (p < 0.001) by 27.3%. Subsequently, at 25 and 50 µg/ml, it reduced (p < 0.05) infectivity to 76.3% and 50.3%, respectively. The intracellular replication of parasites in infected cells considerably decreased (p < 0.001) after treatment with various concentrations of CLE (**Figure 3**).

### Effect of CLEO on NO production

**Figure 4:**
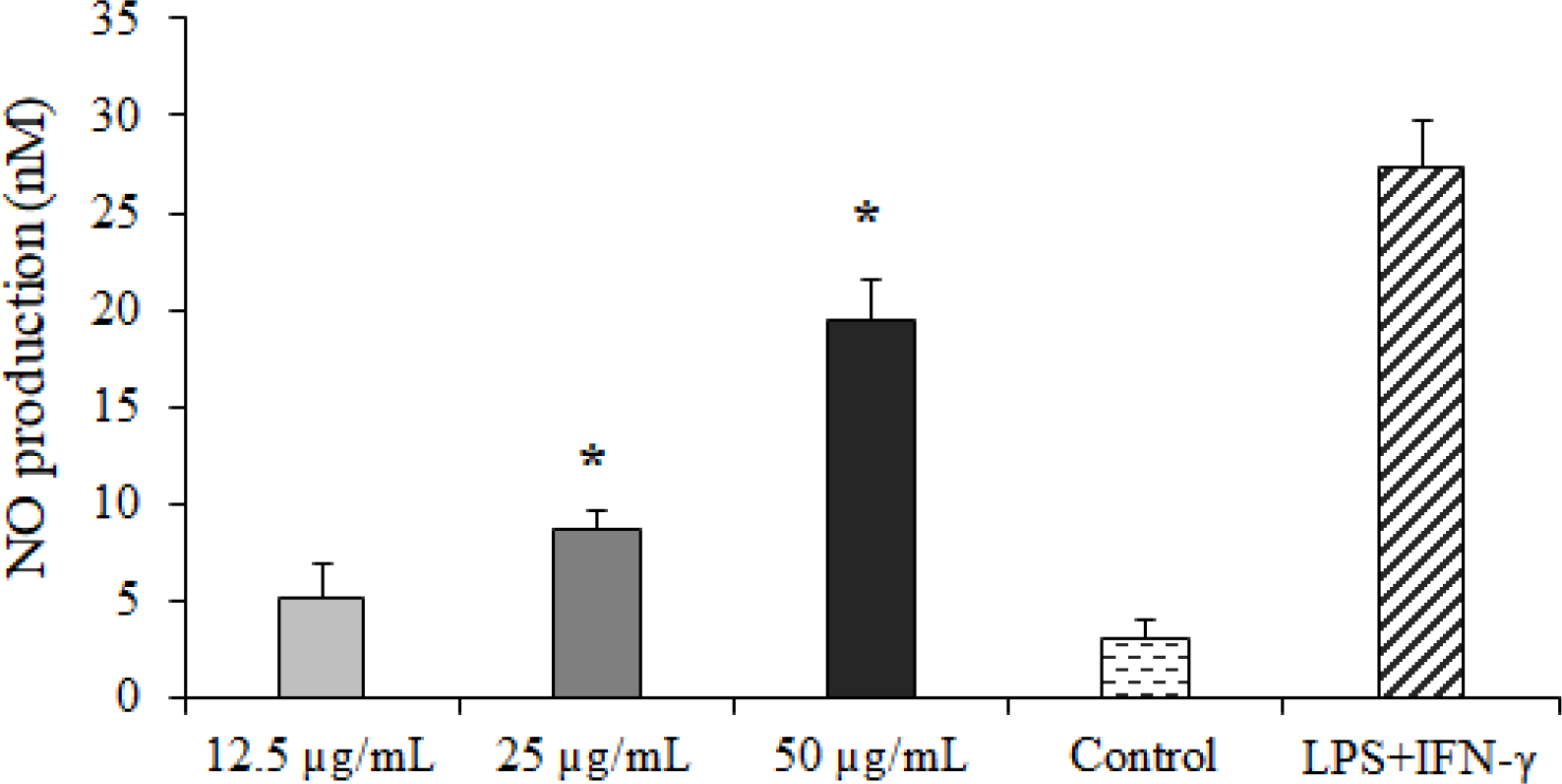
The effect of *Curcuma longa* essential oil (CLE) at 12.5, 25, and 50 µg/ml on nitric oxide (NO) production. *p < 0.001 compared with the non-treated control.

The findings of the Griess reagent assay exhibited that CLEO induced NO production in a dose-dependent manner (**Figure 4**), whereas a significant difference was reported at the concentration of 25 and 50 µg/ml compared to the non-treated cells.

### *In vivo* activity on toxoplasmosis in mice

**Figure 5:**
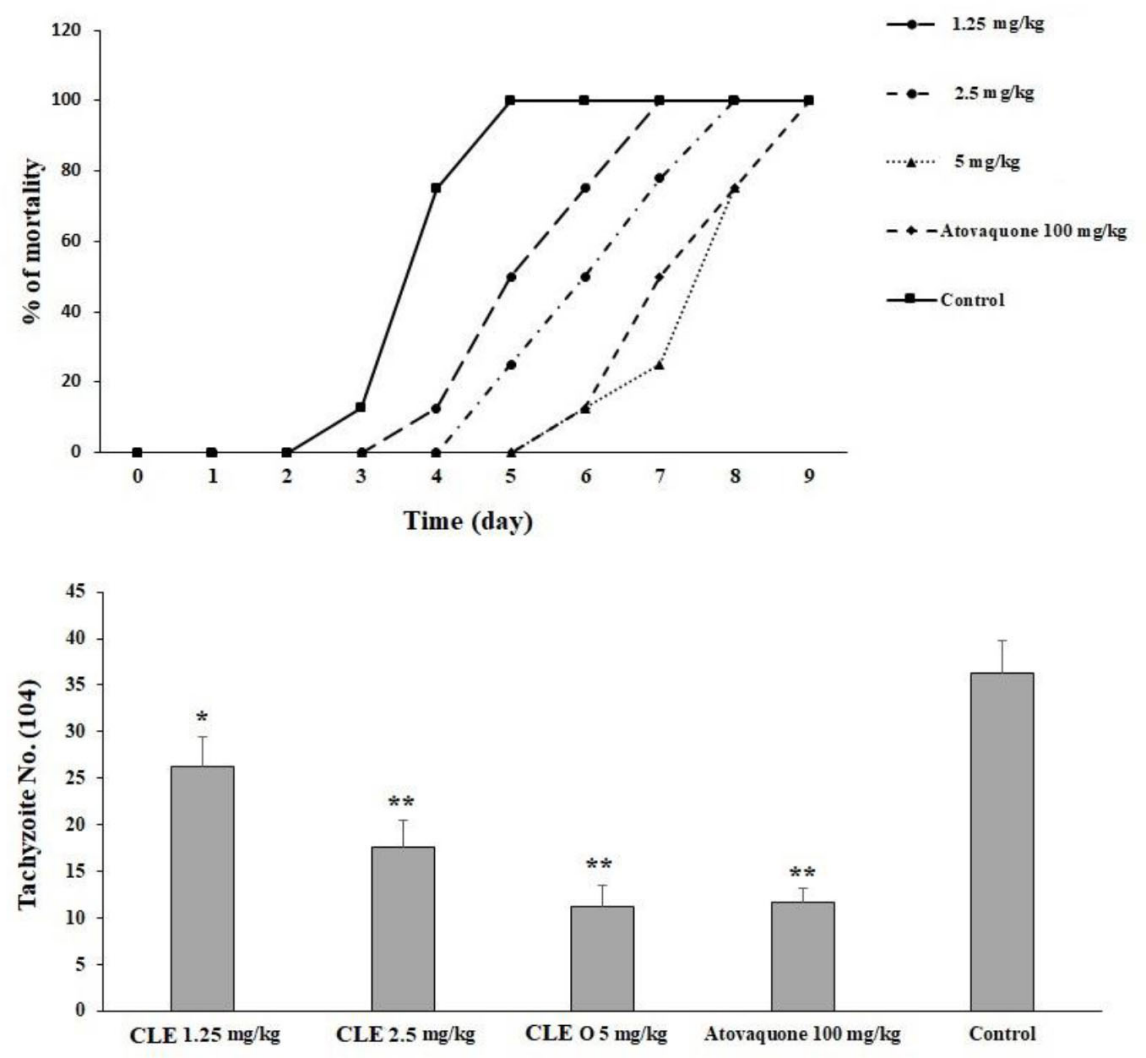
The mortality rate of infected mice pre-treated with *Curcuma longa* essential oil (CLE) at the doses of 1.25, 2.5, and 5 mg·kg^−1^·day^−1^ for 14 days in comparison with the atovaquone 10 mg/kg and control group. Data are expressed as the mean ± SD. The mean number of tachyzoites in the infected mice pre-treated with CLE at the doses of 1.25, 2.5, and 5 mg·kg^−1^·day^−1^ for 14 days in comparison with the control group. Data are expressed as the mean ± SD. *p < 0.05; **p < 0.001.

In the infected mice pre-treated with CLE at 1.25, 2.5, and 5 mg·kg^−1^·day^−1^ for 2 weeks, the survival rate increased by the seventh, eighth, and ninth days, respectively. The rate of the peritoneal parasites considerably declined (p < 0.05) by 72.4%, 48.5%, and 31.1% after receiving CLE at 1.25, 2.5, and 5 mg·kg^−1^·day^−1^, respectively. However, the rate of peritoneal parasites in mice receiving atovaquone 100 mg/kg declined to 32.3% (**Figure 5**).

### Evaluation of oxidative stress and antioxidant enzymes

**Figure 6:**
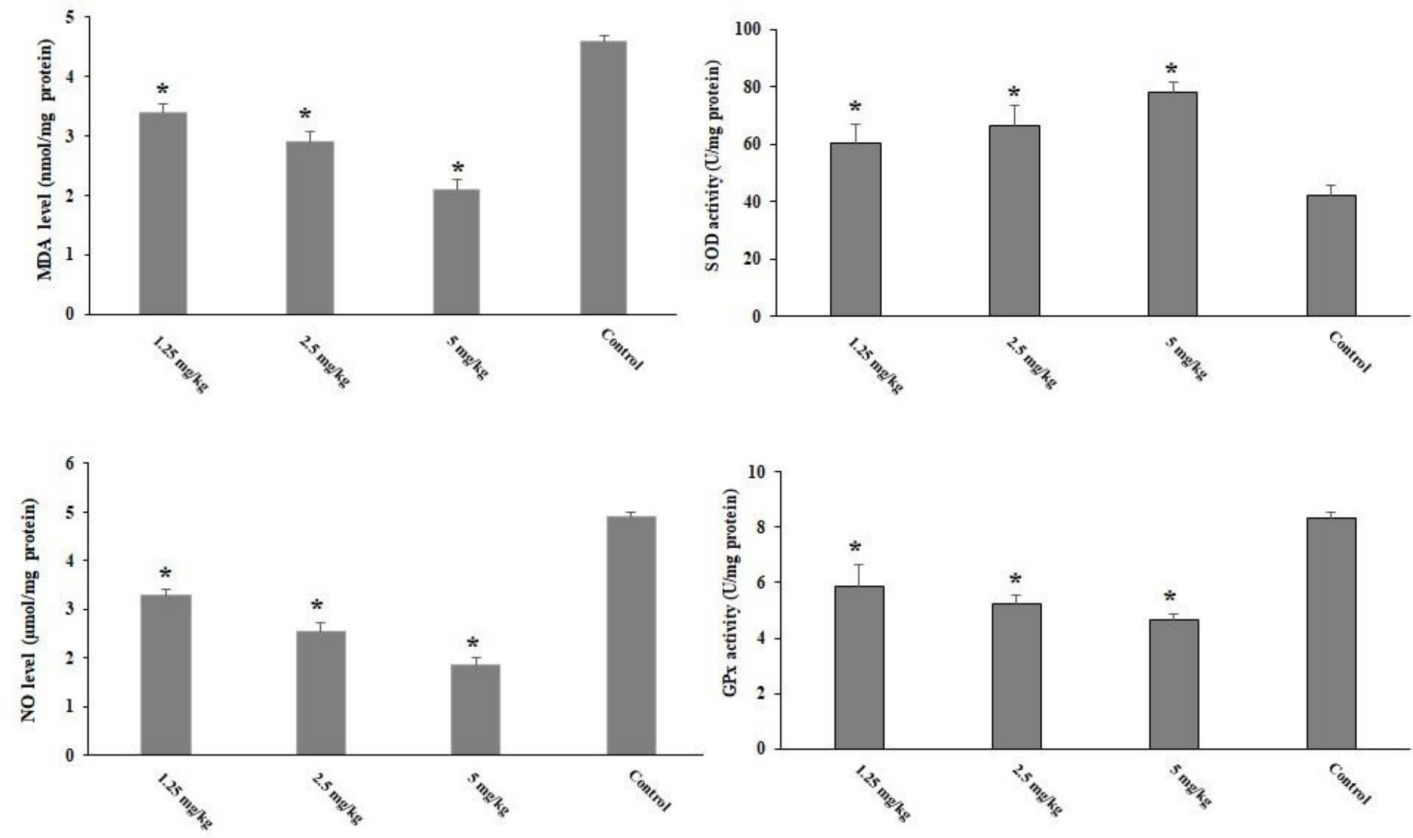
The level of hepatic malondialdehyde (MDA), nitric oxide (NO), glutathione peroxidase (GPx), and superoxide dismutase enzyme activity (SOD) in the *Toxoplasma gondii*-infected mice pre-treated with *Curcuma longa* essential oil (CLE) at the doses of 1.25, 2.5, and 5 mg·kg^−1^·day^−1^ in comparison with the control group. Data are expressed as the mean ± SD (n = 6). *p < 0.001.

Although *T. gondii* infection increased the levels of liver malondialdehyde (MDA) and NO in the animals, in the infected mice pre-treated with CLE for 2 weeks, oxidative stress levels were significantly (p < 0.05) controlled by reducing the levels of MDA and NO. Toxoplasmosis also decreased the levels of GPx and SOD, whereas in the infected mice pre-treated with CLE for 2 weeks, the levels of GPx and SOD significantly (p < 0.05) increased compared with the control group (**Figure 6**).

### Measuring proinflammatory cytokines

**Figure 7:**
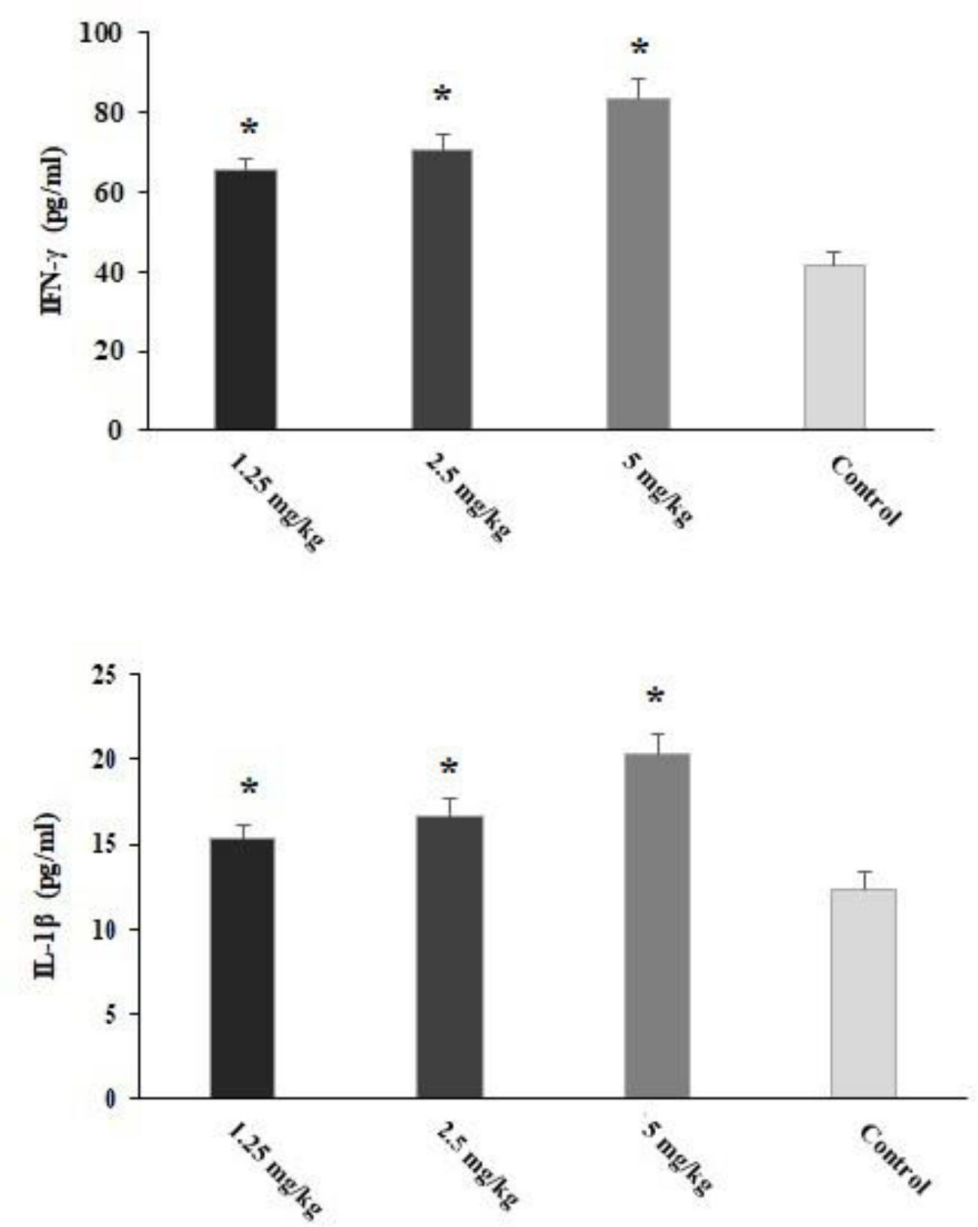
The level of IFN-γ and IL-1β in the *Toxoplasma gondii*-infected mice pre-treated with *Curcuma longa* essential oil (CLE) at the doses of 1.25, 2.5, and 5 mg·kg^−1^·day^−1^ in comparison with the control group. Data are expressed as the mean ± SD. *p < 0.05 (n = 6).

The infected mice pre-treated with CLE for 2 weeks showed significantly (p < 0.05) increased levels of IFN-γ and IL-1β cytokines on the third day post-infection (**Figure 7**).

### mRNA expression level of cytokines

**Figure 8:**
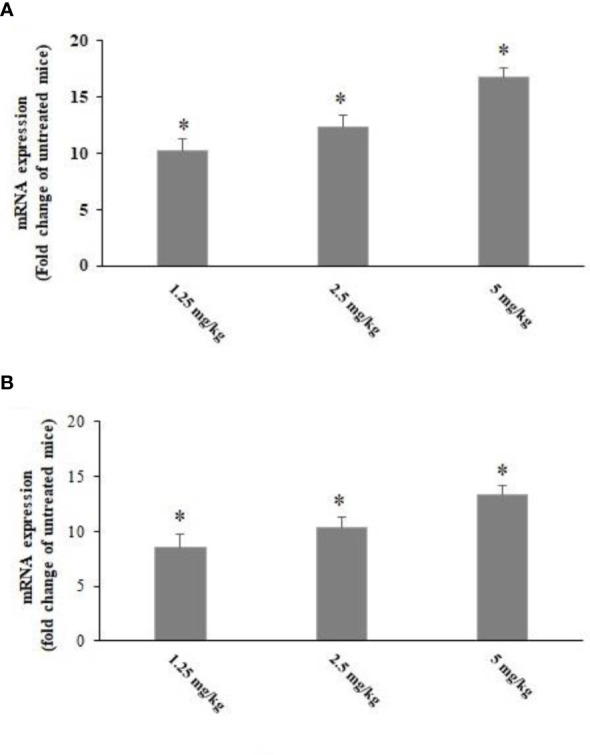
The expression level of IFN-γ **(A)** and IL-1β **(B)** mRNA in the *Toxoplasma gondii*-infected mice pre-treated with *Curcuma longa* essential oil (CLE) at the doses of 1.25, 2.5, and 5 mg·kg^−1^·day^−1^ in comparison with the control group. Data are expressed as the mean ± SD (n = 6). *p < 0.001.

Based on the results of the 2^−ΔΔCT^ method in real-time PCR, the *T. gondii*-infected mice pre-treated with CLE for 2 weeks showed a significant (p < 0.001) upregulation of IL-1β and IFN-γ mRNA genes (**Figure 8**).

### Toxicity properties of CLE in vital organ function

**Figure 9:**
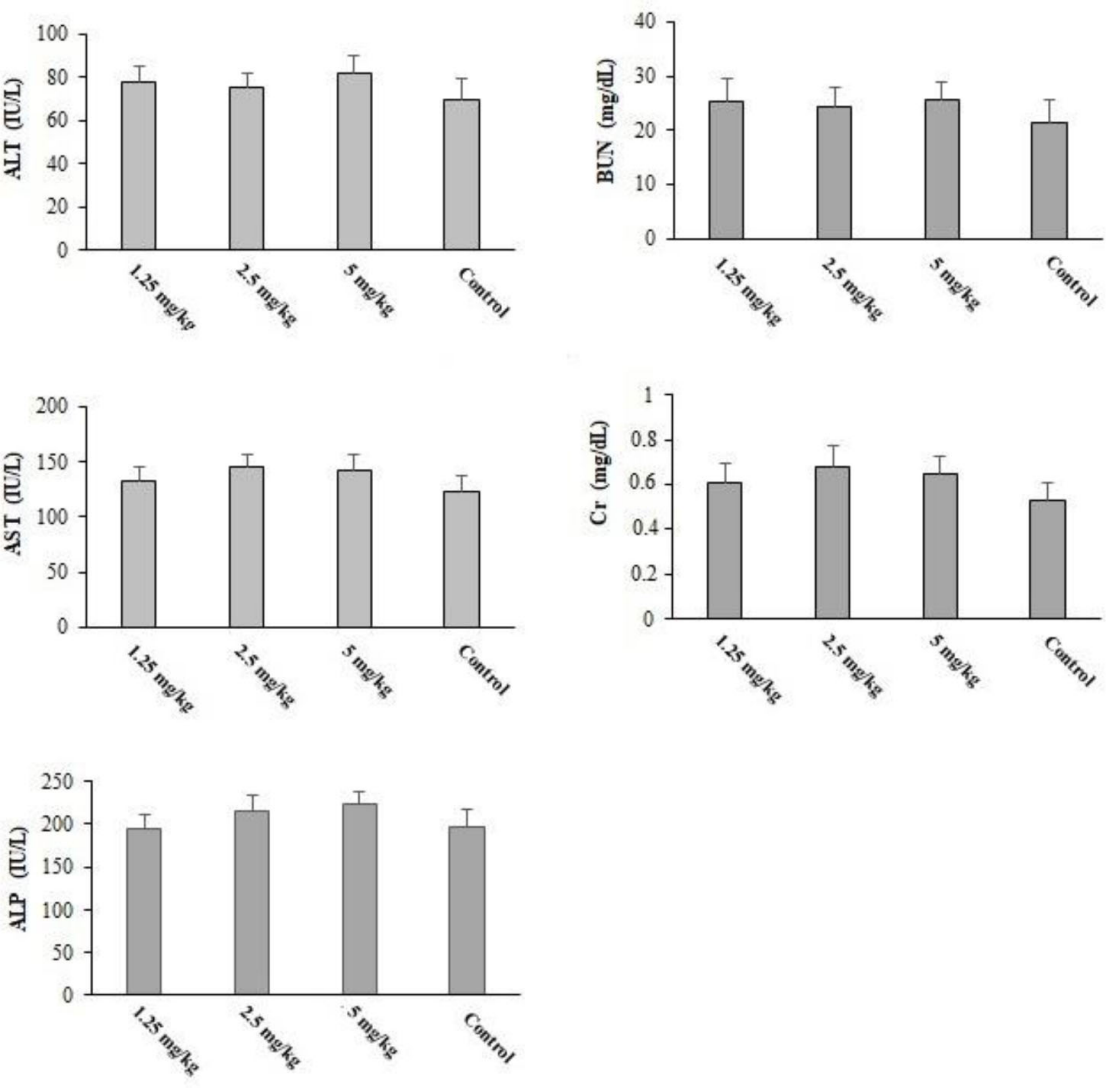
The serum level of some biochemical factors after oral administration of *Curcuma longa* essential oil (CLE) at the doses of 1.25, 2.5, and 5 mg·kg^−1^·day^−1^ for 14 days. ALT, alanine aminotransferase; AST, aspartate aminotransferase; ALP, alkaline phosphatase; Cr, creatinine; BUN, blood urea nitrogen. Data are expressed as the mean ± SD.

Pre-treatment with CLE at 1.25, 2.5, and 5 mg·kg^−1^·day^−1^ for 2 weeks induced no death in the tested mice. Additionally, the results of biochemical examinations revealed no major difference in the serum level of liver and kidney enzymes in comparison with the control group (**Figure 9**).

## Discussion

In recent years, due to a lack of an effective vaccine, effective prophylactic strategies for toxoplasmosis, especially in pregnant women and/or immunocompromised patients with a CD4 < 100 cells/μl, have been strongly recommended (Dubey et al., 2012).

In this study, CLE demonstrated significant (p < 0.001) *in vitro* inhibitory effects on the growth rate of parasites after 0.5–3 h of incubation when compared with the control group. CLE at 25 and 50 µg/ml caused 100% mortality in tachyzoites after 3 and 2 h of incubation, respectively. The intracellular replication of parasites in infected cells considerably decreased (p < 0.001) following treatment with various concentrations of CLE. Based on the *in vivo* assay, the infected mice pre-treated with CLE at 1.25, 2.5, and 5 mg·kg^−1^·day^−1^ for 2 weeks showed an increased survival rate and fewer peritoneal parasites.

Considering the antiparasitic effects of CLE, Mahmudovand et al. (2019) reported that CLE has potent proto-scolicidal effects on *Echinococcus granulosus* protoscoleces; at 50 and 100 μl/ml, it showed the best efficiency (Albalawi et al., 2021). Magalhães et al. reported that this herb has significant *in vitro* effects at doses of 20 to 100 μM on adult *Schistosoma mansoni* worms and considerably reduces egg production (Shakibaie et al., 2013). Recently, in a study by Nasai et al. (2016), it was proved that the ethanolic extract of turmeric has significant effects on the L3 larvae of nematode *Haemonchus* when compared to levamisole (Mahmoudvand et al., 2019).

Based on our findings, the main components were β-turmerone (21.8%), Ar-turmerone (14.7%), and α-turmerone (12.4). In previous studies, the principal constituents of CLE were Ar-turmerone and turmerone (Niranjan and Prakash, 2008). However, the chemical structure of essential oils depends on several factors, e.g., climatic conditions, harvest season, and the part used, which may affect the biological features of herbs (Saedi Dezaki et al., 2016).

As for the antimicrobial mechanisms of action of oxygenated sesquiterpenes such as turmerone and Ar-turmerone, previous studies have reported that these ingredients display their antimicrobial mechanisms through cell wall destruction and by disrupting the vital intracellular. Consequently, it can be suggested that the considerable *in vitro* anti-*Toxoplasma* activity of CLE is due to the existence of oxygenated sesquiterpenes such as turmerone and Ar-turmerone.

One of the most important mechanisms involved in liver injury pathogenesis is LPO during the parasitemia stage of toxoplasmosis. It has been proven that LPO is a marker of oxidative stress (Souza et al., 2017; Nolan et al., 2018). Here, we found that infected mice pre-treated with CLE for 2 weeks showed significantly controlled oxidative stress by a reduction in the level of MDA and NO, as well as considerably improved levels of GPx and SOD. A previous study confirmed the protective efficacy of CLE against induced hepatotoxicity in rats by decreasing hepatic MDA production and increasing glutathione *S*-transferase (GSH), catalase (CAT), SOD, and GPx (Lee et al., 2010). Therefore, it can be claimed that CLE, through its anti-inflammatory and antioxidant effects, delayed the liver damage induced by *T. gondii*. Based on prior investigations, one of the main factors that can prevent infection and improve the host’s survival is the production of some cellular mediators and proinflammatory cytokines IL-1β, IFN-γ, etc (Souza et al., 2017). Here, we found that infected mice pre-treated with CLE for 2 weeks showed a notable upregulation of IL-1β and IFN-γ mRNA genes, indicating that CLE probably increased the host’s survival by boosting the immune system.

It has been previously shown that the NO-related cytotoxic effects provoked by activated macrophages and induction of apoptosis or programmed cell death are the principal mechanisms for eliminating intracellular pathogens such as *Toxoplasma* (Scharton-Kersten et al., 1997; Besteiro, 2015). We found that CLE dose dependently increased the number of the apoptotic cells and induced NO production in a dose-dependent manner in macrophage cells, indicating that triggering the NO and apoptosis induction are the main mechanisms of CLE for controlling *T. gondii* parasites in host cells.

We found that pre-treatment with CLE at 1.25, 2.5, and 5 mg·kg^−1^·day^−1^ for 2 weeks displayed no death and no important difference in the serum level of liver and kidney enzymes in comparison with the control group; this suggests that CLE at the tested doses has high efficacy for toxoplasmosis with minimum toxicity in mice.

## Conclusion

The findings of this study confirmed the significant *in vitro* inhibitory effects of CLE on *T. gondii* tachyzoites of CLE against the *T. gondii* Rh strain. Furthermore, the results exhibited the promising *in vivo* effects of CLE as it improved the survival of infected mice and decreased the parasite count in them. Although the mechanisms of action of CLE are not clear, our study revealed that this may have occurred by promoting the innate immune system and proinflammatory cytokines, reducing oxidative stress, inhibiting hepatic injury, etc. Still, more studies are required to confirm these results.

## Data availability statement

The original contributions presented in the study are included in the article/**Supplementary Material**. Further inquiries can be directed to the corresponding author.

## Ethics statement

The animal study was reviewed and approved by Sirjan Faculty of Medical Sciences, Kerman, Iran (IR.SIRUMS.REC.1400.007).

## Author contributions

FE: Study design and experiments. HM: critical review and validation. YR: Writing the draft and validation. All authors contributed to the article and approved the submitted version.
